# Endocarditis in Cattle Caused by *Bartonella bovis*

**DOI:** 10.3201/eid1309.070236

**Published:** 2007-09

**Authors:** Renaud Maillard, Elisabeth Petit, Bruno Chomel, Caroline Lacroux, François Schelcher, Muriel Vayssier-Taussat, Nadia Haddad, Henri-Jean Boulouis

**Affiliations:** * Ecole Nationale Vétérinaire d’Alfort, Maisons-Alfort, France; † Institut National de la Recherche Agronomique, Maisons-Alfort, France; ‡ University of California, Davis, California, USA; § Ecole Nationale Vétérinaire de Toulouse, Toulouse, France

**Keywords:** *Bartonella bovis*, cattle, endocarditis, dispatch

## Abstract

This study aimed to determine the role of *Bartonella* as an endocarditis agent in cattle. *Bartonella bovis* was identified by PCR, gene sequences analysis, and specific internal transcribed spacer amplicon product size in 2 bovine endocarditis cases with high antibody titers, which demonstrates that *B. bovis* is a pathogen for cattle.

Bacteria-induced vegetative valvular endocarditis is one of the main cardiac disorders in adult cattle (*1*). The prevalence of endocarditis may reach 5.2 cases per 10,000 cows (*2*), but the disease is often misdiagnosed and only discovered during the slaughtering process or at necropsy. Bacterial endocarditis is often linked to a primary source of infection and the presence of other infectious lesions, such as mastitis, metritis, arthritis, or liver abscesses. The most frequent pathogens isolated from cardiac valves or the bloodstream of cows with endocarditis are *Arcanobacterium pyogenes* (up to 90% of the strains), *Streptococcus* sp., and numerous *Enterobacteriaceae* (*2*).

The development of molecular techniques (PCR) led to the identification of many noncultivable or poorly cultivable bacteria as agents of human endocarditis, such as *Coxiella burnetii,* different *Bartonella* species, or *Tropheryma whipplei* (*3*,*4*). In dogs, *Bartonella* species cause 8% of all bacterial endocarditis and up to 19% of noncultivable bacterial endocarditis (*5*). In cats, bacterial endocarditis is infrequent, but a fatal case caused by *B. henselae* was recently reported (*6*). Bartonellae can therefore be considered as potential agents of endocarditis even in their own reservoir species. Cattle host *B. bovis*, which has also been isolated from cats (*7*) and was recently suggested as the etiologic agent in a human case of bartonellosis (*8*). However, the role of *Bartonella* species in bovine endocarditis has never been explored. Therefore, our objective was to determine the putative role of *Bartonella* sp. as an agent of endocarditis in cattle.

## The Study

Twenty-two cases of bovine endocarditis were diagnosed in adult cows (ages 5–15 years, mean 7.4 years) at the School of Veterinary Medicine, Toulouse, France, from September 2004 through June 2006. Eighteen cows were hospitalized for poor condition, anorexia, weight loss, wasting syndrome, and abnormal cardiac auscultation. Endocarditis was diagnosed at physical examination. Lesions of the cardiac valves were confirmed at necropsy for all 18 animals. Four additional cases of endocarditis were identified at necropsy after an apparent sudden death. Most of the damaged valves of these 22 animals had large, cauliflower-like lesions.

For each cow, fragments of the vegetative valve and of 1 normal-appearing valve were collected. DNA extraction from each valve sample was performed by using Nucleospin Tissue extraction kit (Macherey-Nagel, Hoerdt, France) according to the supplier’s recommendation.

PCR amplification was performed on all normal-appearing and vegetative valves for the hypervariable V3 zone of the eubacterial 16S rRNA detection, and the 3′ end of citrate synthase gene (*gltA*) *Bartonella* sp. DNA detection. Additional PCR amplification was performed on *gltA-*positive valves for the following *Bartonella-*specific genes or genomic region: *rpoB*, *ribC*, *groEL*, internal transcribed spacer (ITS) of 16S–23S rRNA (*9*,*10*). Amplification products, including those for the 16S rRNA, were subsequently sequenced.

Serology by indirect fluorescent antibody assay (IFA) was performed as reported elsewhere (*11*) on the supernatant extracted from a cardiac blood clot from each cow. Vero cells infected with the type strain of *B. bovis* (CIP 106692^T^), *B. chomelii* (CIP 107869 ^T^), and *B. schoenbuchensis* (NCTC 13165^T^), respectively, were used as antigens.

The 22 vegetative valves included 8 pulmonary valves, 7 tricuspid valves, 6 aortic valves, and 1 mitral valve. The only vegetative mitral valve and 1 of the 6 aortic vegetative valves showed positive results for *Bartonella-*specific *gltA* gene amplification. For both cows, the normal-appearing control valve was PCR negative for this gene. The PCR-positive cows (nos. 04–927 and 05–1406) were the 2 oldest cows (13 and 15 years old, respectively). ITS 16–23 rRNA amplification was obtained only for the damaged valve of cow 04–927; the size of the amplicon product was ≈190 bp, which was identical to the size of the product obtained with the *B. bovis* reference strain (Figure). Amplification of the 16S rRNA and all the other genes studied were PCR positive for the damaged valves. A 16S rRNA PCR amplicon has been obtained from the normal-appearing valve of cow 05–1406 but could not be sequenced. Amplification of all the other genes studied were PCR negative for normal-appearing control valves of both cows. The genes *rpoB* (GenBank accession nos. EF432062, EF432061), *ribC* (accession nos. EF432060, EF432059), *gltA* (accession nos. EF432055, EF432056), *groEL* (accession nos. EF432058, EF432057) were partially sequenced. Sequence identities were respectively 100% with *B. bovis* (*gltA*) and 99% with *B. bovis* (*groEL*, *ribC*, *rpoB*). The sequence obtained with 16S rRNA (accession no. EF432054) had a 99% identity with *B. bovis*.

**Figure:**
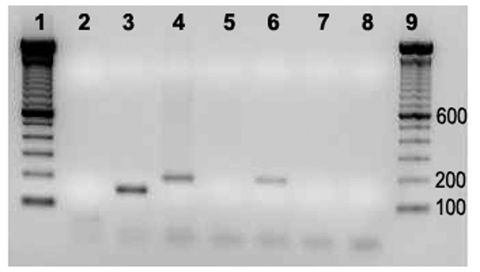
PCR amplification of internal transcribed spacer 16S–23S on vegative and normal-appearing valves of cows 04–927 and 05–1406. 1, Molecular weight marker; 2, negative control; 3, *Bartonella quintana*; 4, *B. bovis*; 5 and 6, normal appearing and vegetative valves (Cow 04–927); 7 and 8, normal appearing and vegetative valves (Cow 05–1406); 9, molecular weight marker.

None of the cultures on rabbit blood agar (*12*) of fragments from the 22 vegetative valves yielded *Bartonella* isolates. The 2 PCR-positive cows had high IFA titers (5,120 and 640 for *B. bovis* antigen), whereas the 20 PCR-negative cows had low or negative titers (Table)

**Table:** Serologic and PCR results for the vegetative heart valves from 22 cows*

Case no.	Age, y	Valve	*Bartonella* PCR (*gltA*)	Indirect fluorescent antibody titer		
				*B. bovis*	*B. chomelii*	*B. schoenbuchensis*
05–1406	13	Aortic	+	5,120	320	640
04–927	15	Mitral	+	640	160	160
05–379	8	Aortic	–	40	–	–
04–269	7	Aortic	–	–	–	–
4071	3.5	Pulmonary	–	40	40	80
3977	5.5	Aortic	–	–	–	–
556	8.5	Pulmonary	–	80	40	–
5988	6	Pulmonary	–	–	–	–
1507	6.5	Tricuspid	–	–	–	–
766	12	Tricuspid	–	–	–	–
4002	5	Tricuspid	–	–	–	–
4815	8	Pulmonary	–	–	–	–
4768	5	Pulmonary	–	–	–	–
4921	6	Tricuspid	–	–	–	–
4784	5.5	Aortic	–	–	–	–
239	10	Pulmonary	–	–	–	–
269	7	Aortic	–	–	–	–
304	12	Pulmonary	–	40	40	40
528	6	Tricuspid	–	–	–	–
975	6.5	Tricuspid	–	–	–	–
1289	10	Tricuspid	–	–	–	–
116	6	Pulmonary	–	–	–	–
379	8	Aortic	–	–	–	–

*gltA, citrate synthase; +, positive; –, negative.

## Conclusions

This is the first description, to our knowledge, of endocarditis associated with *Bartonella* in cattle. PCR amplification of the *gltA* gene, used for identification of *Bartonella* infection, gave an identity of 100% with the previously reported *B. bovis* gene sequence. The sequences of 4 additional genes (*groEL, ribC, rpoB,* and 16S rRNA) shared 99% identity with *B. bovis* genes. ITS amplification of 1 vegetative valve gave a fragment of ≈190 bp, which is the size expected for *B. bovis* (*10*). The lack of PCR amplification of the same genes from healthy-appearing valves indicated that the PCR amplification obtained with the vegetative valves was not the result of a *B. bovis* bacteremia. No definitive evidence exists that *B. bovis* had induced the primary lesion leading to the endocarditis. However, PCR amplification with universal primers for bacterial 16S rRNA allowed us to identify only *Bartonella* sequence in the damaged valves without apparent contamination with DNA from other bacteria.

Moreover, the high IFA antibody titer against *B. bovis* antigen and the low antibody titers of the PCR-negative endocarditis cases reinforced the likely role of *B. bovis* as the causative agent of these 2 bovine endocarditis cases. High antibody titers are commonly observed in human and canine cases of *Bartonella* endocarditis. In fact, high antibody titers are considered a major diagnostic criterion for *Bartonella* endocarditis in humans (*3*). Finally, the 2 *B. bovis*–infected vegetative valves were aortic and mitral valves, which are the most frequently involved valves in human and canine *Bartonella* endocarditis cases (*5*,*13*,*14*).

Two (9.1%) of the 22 endocarditis cases were *Bartonella* DNA positive. This percentage is within the range of 3% reported for human cases of endocarditis (*14*) and 19% (*5*) to 28% (*13*) reported for dogs. The 2 cases occurred in the oldest animals (Table), which suggests that *B. bovis* endocarditis could develop in geriatric cows, following chronic bacteremia in an overtly healthy animal. Nearly 30% of cattle >7 years of age are reportedly *Bartonella* bacteremic (*12*).

Cattle are the main reservoir for *B. bovis,* and diseases attributed to infection with this *Bartonella* species in cows are scarce (*12*). Nevertheless, these 2 cases demonstrated that *B. bovis* is a potential bovine pathogen and that *B. bovis* can induce endocarditis in the animal reservoir host, as previously shown for *B. quintana, B. henselae,* and *B. vinsonii* subsp. *berkhoffii* in humans, cats, and dogs, respectively (*6*,*14*,*15*). This study confirms that *B. bovis* can cause endocarditis in cows like *B. henselae* and *B quintana* in their respective feline and human reservoirs.
